# Parcellation of the Hippocampus According to Its Connection Probability with Prefrontal Cortex Subdivisions in a Malaysian Malay Population: Preliminary Findings

**DOI:** 10.21315/mjms2021.28.3.6

**Published:** 2021-06-30

**Authors:** Aimi Nadhiah Abdullah, Asma Hayati Ahmad, Rahimah Zakaria, Sofina Tamam, Jafri Malin Abdullah

**Affiliations:** 1 Department of Physiology, School of Medical Sciences, Universiti Sains Malaysia, Kubang Kerian, Kelantan, Malaysia; 2 Faculty of Science and Technology, Universiti Sains Islam Malaysia, Negeri Sembilan, Malaysia; 3 Department of Neurosciences, School of Medical Sciences, Universiti Sains Malaysia, Kubang Kerian, Kelantan, Malaysia; 4 Brain and Behaviour Cluster, School of Medical Sciences, Universiti Sains Malaysia, Kubang Kerian, Kelantan, Malaysia; 5 Hospital Universiti Sains Malaysia, Kubang Kerian, Kelantan, Malaysia

**Keywords:** diffusion magnetic resonance imaging, hippocampus, prefrontal cortex

## Abstract

**Background:**

Lesion studies have shown distinct roles for the hippocampus, with the dorsal subregion being involved in processing of spatial information and memory, and the ventral aspect coding for emotion and motivational behaviour. However, its structural connectivity with the subdivisions of the prefrontal cortex (PFC), the executive area of the brain that also has various distinct functions, has not been fully explored, especially in the Malaysian population.

**Methods:**

We performed diffusion magnetic resonance imaging with probabilistic tractography on four Malay males to parcellate the hippocampus according to its relative connection probability to the six subdivisions of the PFC.

**Results:**

Our findings revealed that each hippocampus showed putative connectivity to all the subdivisions of PFC, with the highest connectivity to the orbitofrontal cortex (OFC). Parcellation of the hippocampus according to its connection probability to the six PFC subdivisions showed variability in the pattern of the connection distribution and no clear distinction between the hippocampal subregions.

**Conclusion:**

Hippocampus displayed highest connectivity to the OFC as compared to other PFC subdivisions. We did not find a unifying pattern of distribution based on the connectivity-based parcellation of the hippocampus.

## Introduction

The hippocampus, a medial temporal lobe structure found in all species of mammals, plays a key role in spatial navigation as well as in various modes of learning and memory (1–3). The entorhinal cortex, which connects the hippocampus with the rest of the neocortex, is a main source of its inputs (4). The hippocampus is divided into many subfields including the regions of dentate gyrus and cornum ammonis areas 1 and 3. It is also arranged along a longitudinal axis that stretches from an anterior to a posterior pole in primates, as well as in humans (5). Lesion studies have shown that tasks usually associated with the hippocampus (i.e. the processing of spatial information and memory) are mostly subserved by its dorsal subregion, while the ventral hippocampus is more involved in emotional and motivational behaviours such as anxiety (6). The unique anatomical connections of the dorsal and ventral poles to both afferent and efferent structures also reflect this functional dissociation (7, 8).

The prefrontal cortex (PFC) is a more phylogenetically divergent structure than the hippocampus (4), and it is a critical area for cognitive processes and emotional control of the higher order. The primate PFC is structured into many subregions but can be primarily divided into a dorsolateral division involving cognitive functions such as executive control, attention and working memory, and a ventromedial (or orbitomedial) division more involved in emotional and motivational regulation (9, 10). The PFC receives monosynaptic projections from the hippocampus in both rodents and primates (11–13). These projections originate almost exclusively in the ventral hippocampus and primarily target the medial PFC (mPFC), with some evidence suggesting stronger projections to ventral subregions (11–14). In addition to these monosynaptic connections, bidirectional interactions between the two structures also occur via many indirect routes. One potential relay is the thalamus nucleus reuniens, which is connected reciprocally to both the dorsal and ventral hippocampus as well as the mPFC (15, 16).

Various approaches can be used to study hippocampal-prefrontal interactions in animals and humans. One of them is using diffusion magnetic resonance imaging (dMRI), a method that makes exploration of white matter connectivity in the living brain feasible. The development of dMRI has enabled the evaluation of white matter tracts by virtue of its ability to image water diffusion characteristics (17). In early dMRI studies, deterministic tractography, which estimates only the primary orientation of diffusion in each magnetic resonance imaging (MRI) voxel, was commonly used (17, 18). However, due to crossing fibres, this method was unable to trace all the connected brain regions from the seed voxels (17, 18). In contrast, probabilistic tractography, which is a reflection of the multiple orientations of diffusion and an estimation of more than one fibre population in each MRI voxel, enables tracing of the crossing fibres (17–19).

Accordingly, probabilistic tractography has been widely used for the investigation of the neural connectivity of neural structures in the human brain, including the fornix, lateral geniculate body and red nucleus, to name a few (20–23). To date, a few dMRI studies are available on the anatomical neural tracts between the hippocampus and prefrontal regions (24). However, the possible variability in the structural connectivity between the hippocampus and the six prefrontal subdivisions has yet to be fully explored. In the present study, using probabilistic dMRI tractography, we attempted to track the hippocampal-prefrontal cortex connectivity in healthy Malaysian subjects.

## Methods

### Subjects

Four healthy male subjects with no previous history of neurological, physical or psychiatric illness were recruited for this study. These subjects were healthy controls of another related study involving traumatic brain injury patients. All subjects were right handed, understood the purpose of the study and provided written, informed consent prior to participation. Magnet safety screening was performed prior to scanning.

### Data Acquisition

MRI scan images were acquired using a 3T Philips Achieva MRI scanner (Netherlands) with a 32-channel SENSE head coil. The protocol for the dMRI was as follows: repetition time (TR) = 10,726 ms, echo time (ET) = 76 ms, field of view (FOV) = 221 × 221, matrix = 96 mm × 94 mm, slice thickness = 2.3 mm, 67 slices, *b* = 1,000 s/mm^2^, voxel size of 2.3 × 2.3 × 2.3 mm^3^, and EPI factor = 57 resulting in 32 diffusion weighted volumes (*b* = 1,000 s/mm^2^) and one non-diffusion weighted volume (*b* = 0 s/mm^2^) as reference. T1-weighted image was acquired using the following parameters: TR = 7.4 ms, TE = 3.4 ms, FOV = 250 × 250, matrix size = 228 mm × 227 mm, voxel size = 1.1 mm × 1.1 mm, slice thickness = 1.2 mm and 240 slices. Acquisition of the diffusion imaging data took 7 min per subject.

### Pre-Processing

Data preprocessing utilised tools from FDT (FMRIB’s Diffusion Toolbox), part of FMRIB’s Software Library (FSL version 5.0.9). Diffusion-weighted images were initially corrected for head motion effect and image distortion due to eddy currents. Probability density functions on up to two principal fibre directions were estimated at each voxel in the brain using the Bayesian Estimation of Diffusion Parameters obtained using sampling techniques toolbox (bedpostx; 17) implemented in FSL.

### Definition of Regions of Interest in Structural Space

**Figure 1:**
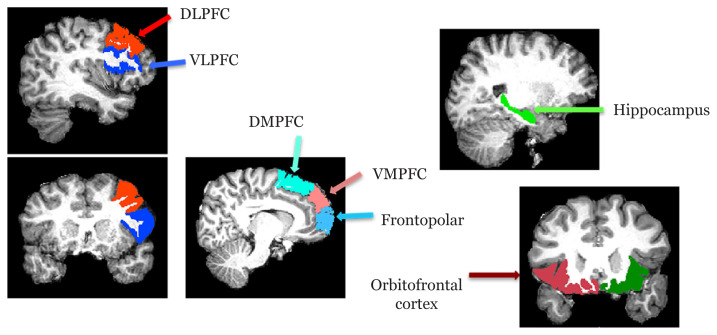
Regions of interest hand-drawn in the structural space of one subject. Seed mask: hippocampus; target masks: six subdivisions of the prefrontal cortex i.e., DLPFC, VLPFC, FPC, OFC, VMPFC and DMPFC

Regions of interest masks were bilaterally hand-drawn on the T1-weighted image (structural space) for each subject according to the anatomical landmarks referred to in the Duvernoy’s atlas of the human brain (25) as shown in Figure 1. The regions of interest included hippocampus as the seed and the six subdivisions of the PFC (Table 1) as the targets, namely the dorsolateral prefrontal cortex (DLPFC), ventrolateral prefrontal cortex (VLPFC), frontopolar cortex (FPC), orbitofrontal cortex (OFC), ventromedial prefrontal cortex (VMPFC) and dorsomedial prefrontal cortex (DMPFC) (24, 26, 27).

### Probabilistic Tractography

Fibre tracking was performed using probtrackx module implemented in FSL, following the method previously described by Behrens et al. (28). The probability distribution of the principal diffusion direction was estimated at each voxel, and the estimated distribution represented uncertainty in the diffusion direction caused by factors that include potential co-existence of many fibre pathways within a single voxel, image noise and subject movement in the scanner (29). The algorithm of probabilistic tractography utilises these local probability distributions to generate streamline samples (fibre pathways) to build up the connectivity distribution in the structural space. Probabilistic tractography was performed using a single-seed approach. The tracking parameters included 5,000 samples per voxel, a step length of 0.5 mm, and a curvature threshold of 0.2. Probabilistic tractography maps were individually generated for the left and right halves of the masks.

### Calculation of Relative Connection Probability

Connection probability, which indicates the probability that a sample initiated from a seed region will reach a particular target region, was obtained from the output of probtrackx. From the 5,000 samples initiated from each voxel in the seed region, the number of samples that reaches the target was multiplied by the number of voxels in the seed area with positive connection probability to the target. The relative connection probability is the percentage of the connection probability for each target over the sum of the connection probabilities to all targets (27, 30).

### Statistical Analysis

Repeated measures ANOVA using SPSS software (v.24.0; SPSS, Chicago, IL) was only used to determine whether there was any differences in the volumes of seed and target masks between the left and right hemispheres. Statistical significance was accepted for *P-*values of less than 0.05.

## Results

### Demographic Data

All subjects were male, age ranged between 21 and 52 years old (mean [SD] = 30.6 [14.4]), right hand dominant, possessed at least nine years of education, had no psychiatric illness and not on any psychiatric drugs (Table 2).

### Region of Interest Mask Sizes

**Figure 2:**
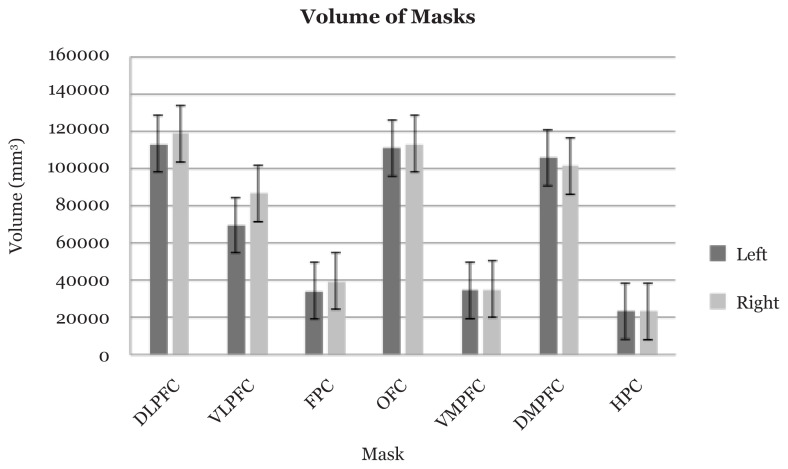
Volumes of seed (hippocampus) and target masks (PFC subdivisions DLPFC), VLPFC, FPC, OFC, VMPFC and DMPFC. Values are mean ± SEM

The comparison of the mask sizes was done to ensure the high accuracy of the mask and uniformity of the mask sizes since the masks were hand-drawn. The different sizes of the seed and target masks are summarised in Figure 2. The biggest mask was the one for the DLPFC (left side, mean [SD] = 28,307.00 [3,626.56]); right side, mean [SD] = 29,659.00 [5,482.86]) and the smallest was the one for hippocampus (left side, mean [SD] = 5,850.50 [668.76]; right side, mean [SD] = 5,815.00 [558.53]). Repeated measures ANOVA with two factors, hemisphere (left and right) and PFC subdivisions (DLPFC, VLPFC, FPC, OFC, VMPFC and DMPFC) showed that the prefrontal masks were not significantly different between hemispheres (main effects of hemisphere: *F*(5, 30) = 0.35, *P* = 0.88) but there was a significant main effect of PFC subdivisions: *F*(5, 30) = 36.18, *P* < 0.001) meaning that the mask sizes for both hemispheres were not significantly different and the differences among the different subregions were as expected.

### Tractography Results

**Figure 3:**
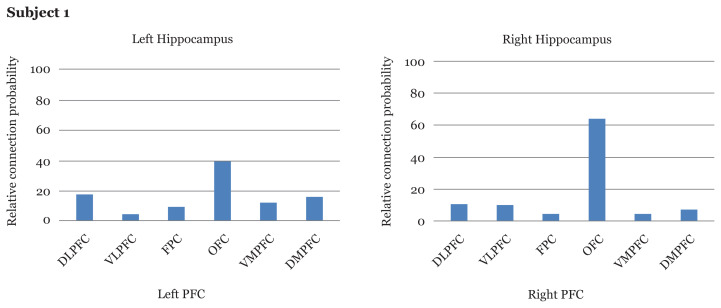

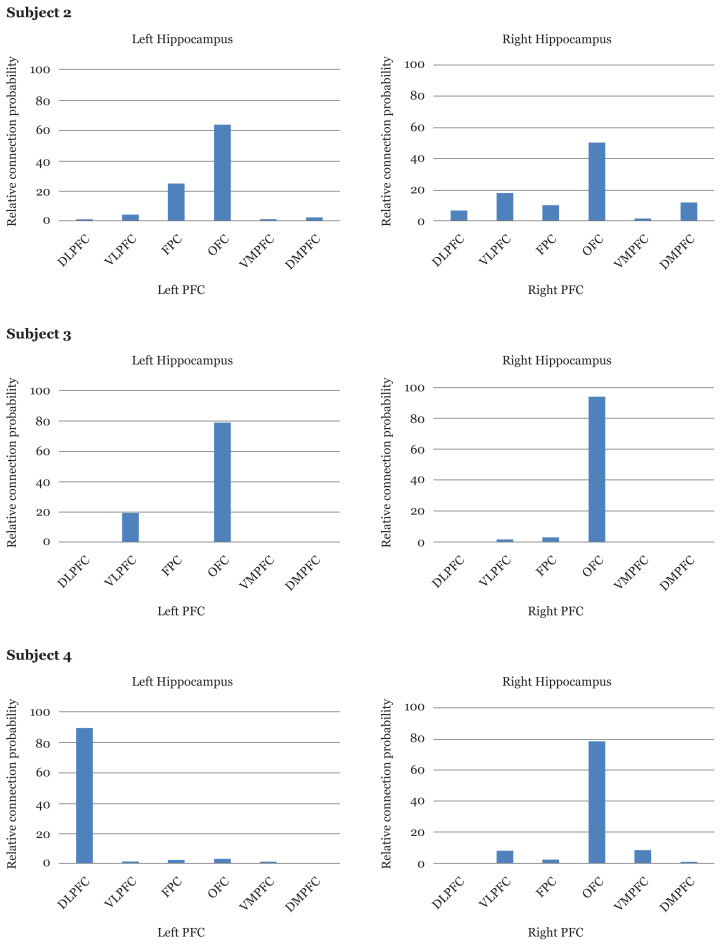
Relative connection probability from hippocampus to six PFC subdivisions on the left and right hemisphere for each subject (*n* = 4)

Tractography was used to compare the relative connection probability between hippocampus and the six subdivisions of the PFC. Individual tracking of the hippocampal-prefrontal tractography revealed variable patterns of connectivity among the subjects and between the right and left hemispheres. In general, the hippocampus showed connectivity to all of the subdivisions of the PFC (i.e., the DLPFC, VLPFC, OFC, FPC, VMPFC and DMPFC). However, there was a high degree of variability among subjects in terms of the pattern of distribution. In general, the highest connectivity was to the OFC in all subjects for both hemispheres (Figure 3).

#### Subject 1

The highest connection probability was to the OFC for both the left and right hemispheres, with the right side displaying higher connection probability as compared with the other PFC subdivisions. Connection probability to the other subdivisions of the PFC was less than 20% each.

#### Subject 2

Similar to subject 1, the highest connection probability was to the OFC. The left hippocampus displayed a relatively lower percentage of connection probability to the other PFC subdivisions except for the FPC.

#### Subject 3

Connection probabilities for both hemispheres were predominantly to OFC, while those to all the other PFC subdivisions were much lower except to the VLPFC over the left hemisphere only.

#### Subject 4

Different from the other subjects, the highest connectivity over the left hemisphere was to the DLPFC with much lower connection probability to all the other PFC subdivisions. Over the right hemisphere, however, the pattern was quite similar to the other subjects with the highest connection probability to the OFC, while the connection probabilities to the other PFC subdivisions were less than 20%.

## Parcellation

**Figure 4:**
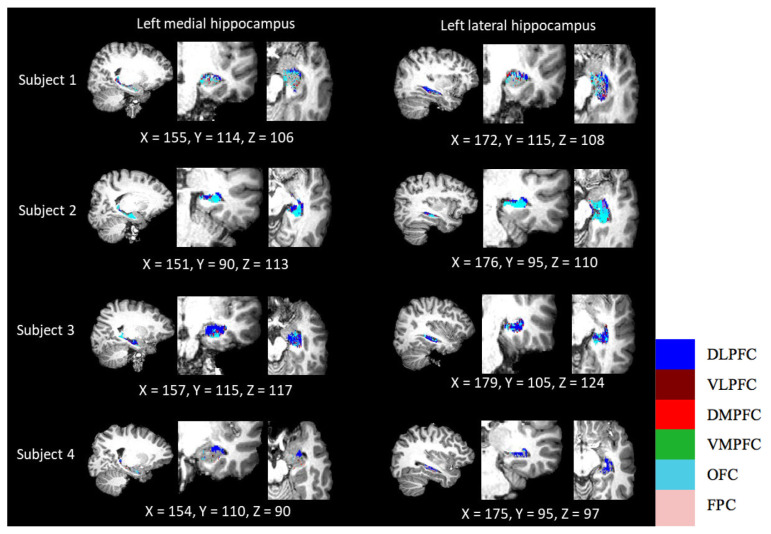
Parcellation of the hippocampus according to its connection probability to six PFC subdivisions in the left hemisphere (*n* = 4)

**Figure 5:**
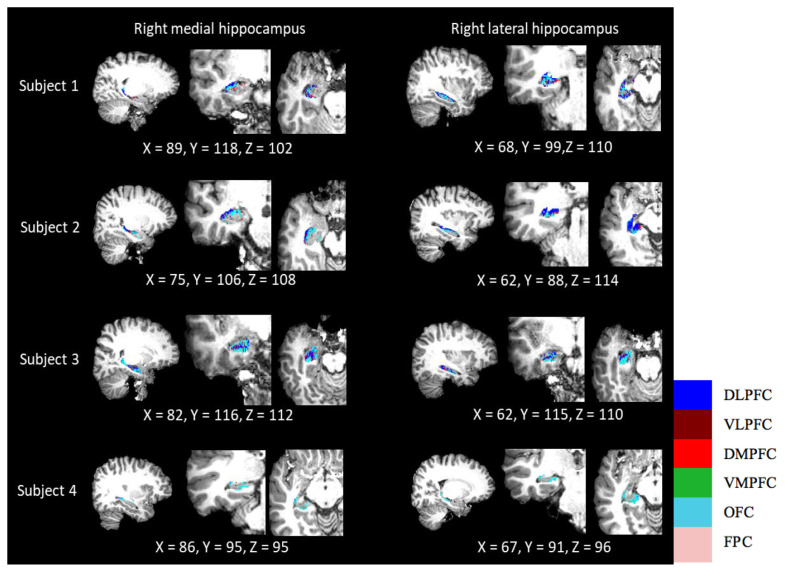
Parcellation of the hippocampus according to its connection probability to six PFC subdivisions in the right hemisphere (*n* = 4)

Parcellation of the hippocampus can indicate where the hippocampus has the highest connection probability with each PFC subdivision. The results showed variable patterns of distribution among the four subjects (Figures 4 and 5). The OFC was visually prominent in most subjects’ hippocampus, either in the medial or lateral view. Another PFC subdivision that featured prominently was the DLPFC, especially in subjects 3 and 4. However, the parcellation maps displayed no distinct pattern that would indicate segregation between the dorsal and ventral hippocampal subregions. Other regions with lower connection probabilities were barely seen in the parcellated map.

## Discussion

Hippocampal-PFC connectivity consists of the main anatomical connection from the hippocampal formation to the PFC, either by monosynaptic or polysynaptic projections, which suggests a crucial role for the hippocampal-PFC circuit in the anatomical and functional coupling of the two regions (31). In the present study, we tracked hippocampal-PFC neural connectivity in four normal healthy human brains using probabilistic tractography.

Based on our findings, the connectivity of the hippocampus was mainly to the OFC of both hemispheres except for subject 4 (left hemisphere). For subject 4, the connectivity of the hippocampus in the left hemisphere was aberrated to the DLPFC region. However, we could not explain the aberration as the present study was conducted in a small sample (*n* = 4) and the analysis used a single-seed approach. A larger sample is preferred for the analysis of variability and ideally a network is constructed from the individual-based parcellation to better extract the variability from true subject difference.

The strong connectivity to the OFC may be explained by the similar functional roles of hippocampal formation and the OFC (32). Historically the hippocampus has been associated with mapping (33) and later, its role in encoding information about the world in a way that facilitates flexible and inferential cognitive processes has been put forward (1, 2, 34–37). The OFC, by comparison, has traditionally been related to reward- and value-based behaviours (38–45). However, it has recently been suggested that the OFC may have a cognitive-map-like function. Thus, it has been proposed that a fundamental function of the OFC is to form and to maintain neural representations of task state, that is, a representation of all the relevant internal and external stimuli or features that define a particular situation in the world (46, 47). Because this function requires the OFC to encode both features of the environment (including observable sensory properties and unobservable, implicit variables that must be inferred) and how relationships between those features might change in different situations, the OFC has been described as a cognitive map of task state (47). From this view point, the OFC and hippocampus each contribute to cognitive mapping and the resultant behaviour (32).

Connectivity-based parcellation of the hippocampus according to its probabilistic connectivity to the PFC subdivisions in the four subjects studied were highly variable and produced no unifying pattern among them and did not conform to any established structural subregions of the hippocampus (48). This may suggest that the segmentation of the hippocampus does not follow its structural connectivity to PFC subdivisions. However, the small sample used does not make this conclusive. A recent study performed the functional parcellation of the hippocampus into head, body and tail parcels and found that functional parcellation did not strictly follow structural parcellation (49).

Several limitations of this study should be considered. First, as mentioned earlier, a larger sample size is required to confirm the present findings. Second, the fact that the hippocampus is composed of several regions, future studies should be conducted to find the connectivity from specific regions of the hippocampus to each PFC subdivision. Third, the use of probabilistic tractography can result in false positive and negative findings due to fibre complexity or partial volume effects throughout the white matter of the brain (54, 55). Fourth, tractography assumes monosynaptic connectivity and provides little or no information on indirect connectivity. Therefore, our results is to be read with caution.

## Conclusion

In general, our preliminary findings indicate that in normal healthy subjects, the hippocampus showed the highest connectivity to the OFC as compared to other PFC subdivisions. We did not find a unifying pattern of distribution based on the connectivity-based parcellation of the hippocampus nor any indication that the connectivity follows structural subregions.

## Figures and Tables

**Table 1:** Subdivisions of PFC according to Brodmann areas (BA)

No	PFC subdivisions	BA
1	DLPFC	BA 8, 9, 46 and 9/46 in the superior and middle IFG (28)
2	VLPFC	BA 44 (pars opercularis), 45 (pars triangularis) and lateral part of area 47/12 of IFG (28, 29)
3	FPC	BA 10 (30)
4	OFC	BA 10, 11, 12, 13, 14 and orbital part of area 47/12 (29, 31)
5	VMPFC	BA 10, 14, subgenual cingulate cortex (BA 32 and 25) and ventral ACC (32)
6	DMPFC	Medial regions of BA 8, 9 and 9/46 (30)

**Table 2:** Participants’ particulars

Participant	Age (years old)	Education (years)
1	26	17
2	52	11
3	21	14
4	23	16
